# Relationship between Moisture Transportation, Efflorescence and Structure Degradation in Fly Ash/Slag Geopolymer

**DOI:** 10.3390/ma13235550

**Published:** 2020-12-05

**Authors:** Shuaikang Zhou, Suhua Zhou, Jiuchang Zhang, Xin Tan, Deng Chen

**Affiliations:** 1 College of Civil Engineering, Hunan University, Changsha 410082, China; Zhou0328@hnu.edu.cn (S.Z.); zhousuhua@hnu.edu.cn (S.Z.); 2 Key Laboratory of Building Safety and Energy Efficiency of the Ministry of Education, Hunan University, Changsha 410082, China; 3 Faculty of Civil Engineering and Mechanics, Kunming University of Science and Technology, Kunming 650500, China; zhangjiuchang@foxmail.com; 4 College of Civil Engineering, Suzhou University of Science and Technology, Suzhou 215011, China; chendeng0310@gmail.com

**Keywords:** efflorescence, geopolymer, moisture transportation, pore structure

## Abstract

The relationship between moisture transportation and efflorescence in sodium hydroxide- or sodium silicate-activated fly ash/slag geopolymers was investigated. The results show that the efflorescence products are sodium carbonate hydrates, mainly composed of natron, heptahydrate, trona and sodium carbonate. The efflorescence induces compressive strength loss, water absorption increases and pore structure degradation in the geopolymer. When the curved surface of a geopolymer cylinder is covered with plastic film, the moisture transportation drives the free alkalis to the top surface to initiate efflorescence. In comparison, the efflorescence occurring on the curved surface of an uncovered geopolymer cylinder results in a more intensive alkalinity loss. For the uncovered geopolymers prepared with sodium hydroxide activator, efflorescence deposits are formed on the lower half of cylinder. A low capillary absorption capacity developed in the pore structure can only drive the moisture to the middle of cylinder, which is confronted with the drying front. More efflorescence products are formed on the upper half of the uncovered geopolymer cylinder prepared with sodium silicate activator. A relatively higher capillary absorption capacity, developed in the more compact pore structure, transports the moisture from the bottom to the top of cylinder, so no drying line is observed in the cylinder.

## 1. Introduction

Geopolymer is prepared by alkali-activation of solid aluminosilicate precursors, commonly including metakaolin, fly ash and blast furnace slag, or their blends [1,2]. The alkali hydroxide and silicate or their concentrated solutions are used as the alkali activators, which supply required alkalinity for initiating the dissolution of aluminosilicate precursors and the following geopolymerization [3]. However, the extremely high pH environment and high availability of OH^−^ and Na^+^ ions in geopolymers are liable to cause some durability issues, especially for efflorescence [4,5]. Efflorescence is a series of physiochemical carbonation processes frequently occurring on the drying surface of geopolymers [4]. Water evaporation that occurs on the exterior of geopolymer specimens induces moisture transportation in the pore structure, which moves the free alkalis from the matrix to the surface to react with the atmospheric CO_2_ [6,7]. The white carbonation deposits are mainly composed of various types of sodium carbonate hydrates [8,9]. 

It is generally accepted that efflorescence severely influences the appearance of geopolymer products. Recent studies also indicate that efflorescence induces structural degradation, because the continuous alkali leaching decreases the pore solution alkalinity in the geopolymer [6,9]. Although a detectable phase change is not observed in the geopolymers with intensive efflorescence, this carbonation process can be assigned to a neutralization process for consuming OH^−^ and Na^+^ ions preserved in the geopolymer network [9]. Najafi Kani et al. [5] put forward a close relationship between the alkali leaching due to efflorescence and strength reduction in geopolymers based on natural pozzolan. Zhang et al. [9] observed that the subflorescence under the surface of geopolymer generated crystallization pressure in the pore structure. Strength loss due to efflorescence was also observed in the geopolymer pastes prepared with fly ash and slag blends [6].

Efflorescence deposits are rapidly formed on the surface of geopolymer products when the other sides are in contact with water [4]. The wetting or drying conditions used for curing the geopolymers play dominant roles in controlling the moisture absorption/desorption isotherms in the matrix [10]. In addition, the pore structure also correlates well with the moisture transportation, which significantly influences the movement of free alkalis in the pore network [11,12]. Babaee and Castel [13] investigated the relationship between moisture transportation and pore structure in the geopolymer and Portland cement pastes. It was proposed that the pore size diameter, total porosity and tortuosity of pore networks significantly affected the moisture transportation [13,14,15,16]. Longhi et al. [8] explored the effective methods of mitigating efflorescence in metakaolin-based geopolymers and observed that the addition of soluble silicates in activator or thermal curing (50 °C) could compact the pore structure to restrict the free alkalis leaching in the matrix. Blast furnace slag addition also compacted the pore structure of fly ash-based geopolymers to mitigate the efflorescence [6,7]. 

An improved knowledge on the relationship between the moisture transportation, efflorescence development and structure degradation is a key requirement to understand the efflorescence mechanism in geopolymers, especially in the products used for field applications. This is helpful in proposing more efficient methods of mitigating efflorescence. Therefore, this research investigates the effects of moisture transportation on the alkali leaching, compressive strength development and water absorption of geopolymers after different efflorescence curing regimes. The structure degradation of geopolymers is characterized by using the nitrogen adsorption technique and a scanning electron microscope.

## 2. Experimental 

### 2.1. Materials

**Figure 1:**
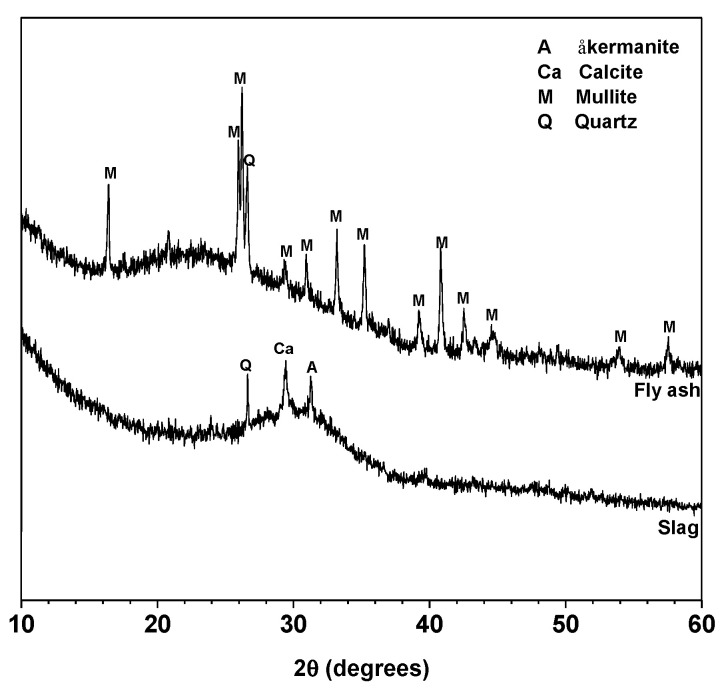
XRD patterns of fly ash and slag. Phases identified: åkermanite (A), Ca_2_MgSi_2_O_7_, PDF No. 74-990; Calcite (Ca), CaCO_3_, PDF No. 83-1762; Mullite (M), Al_5.65_Si_0.35_O_9.175_, PDF No. 82-1237 and Quartz (Q), SiO_2_, PDF No. 74-1811.

Class F fly ash and granulated blast furnace slag were obtained from Xuzhou Guohua Power Station (Xuzhou, China) and Wuhu Hailuo Cement Corporation (Wuhu, China), respectively. The chemical compositions determined by X-ray fluorescence (XRF) are shown in Table 1. The XRD patterns of fly ash and slag are shown in Figure 1. Both fly ash and slag are mainly amorphous. Mullite and quartz are the main crystalline phases in fly ash, and åkermanite, quartz and calcite are the crystalline phases in slag. The mean particle sizes (*D*_50_) of fly ash and slag were tested by using Malvern Masterizer 2000 laser particle analyzer (Malvern, UK), and the values are 14.71 and 12.31 μm, respectively.

The pore solution chemistry (e.g., alkalinity and ions concentrations) is significantly influenced by the activator type used for preparing geopolymers. To better understand the efflorescence mechanism, two types of activators were used in this work, including an NaOH solution and a sodium silicate solution. The 8 mol/L NaOH solution was prepared by dissolving NaOH pellets (96% purity) in the deionized water. The sodium silicate solution was prepared by dissolving commercial sodium silicate solution (SiO_2_ = 32.6 wt.%, Na_2_O = 10.4 wt.%) and NaOH pellets in the water to achieve an SiO_2_/Na_2_O molar ratio of 1.2. The (Na_2_O + SiO_2_) solids concentration in the sodium silicate activator was set as 31.3% to ensure this activator had the same alkalinity (OH^−^ ions concentration) as the 8 mol/L NaOH solution.

### 2.2. Synthesis of Geopolymer Pastes

In total, 80 wt.% fly ash and 20 wt.% slag were dry-mixed firstly, and then blended with the activator at a liquid to solid ratio of 0.50 to prepare the geopolymer pastes with a good workability. The slag addition in the fly ash-based geopolymer pastes ensured early-age rapid strength development at room temperature. The geopolymers prepared with NaOH and sodium silicate activators were denoted as NHFS and NSFS specimens, respectively. The pastes were cast into the 100 mm diameter × 200 mm height cylindrical mould. After curing at 20 ± 1 °C and relative humidity (RH) ≥ 95% for 24 h, the pastes were demolded and cured for 28 days. Parts of the 28 day-cured samples were further cured at the same conditions for another 28 days and denoted as the control samples.

**Figure 2:**
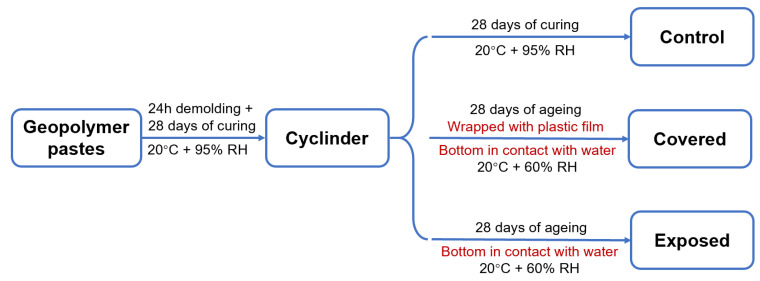
Schematic diagram of the curing regimes.

To better understand the influence of moisture transportation on the efflorescence in the geopolymer cylinder, parts of the 28 day-cured samples were wrapped with plastic film on the curved surface of the cylinder to inhibit the water evaporation, and denoted as the covered samples. The unwrapped geopolymer cylinders were denoted as the exposed samples. The covered and exposed cylinders were placed vertically in a petri dish with the bottom (2 ± 0.5 mm depth) immersed in the deionized water, and subjected to a further 28 day-ageing in a drying curing box (20 ± 1 °C, RH = 60 ± 2%). The water in the petri dish was refilled every 24 h. This ageing regime was used to accelerate the efflorescence degradation. The control, covered and exposed curing regimes are shown in Figure 2. Three replicate samples of each mixture were aged for each regime.

### 2.3. Testing and Characterization

**Figure 3:**
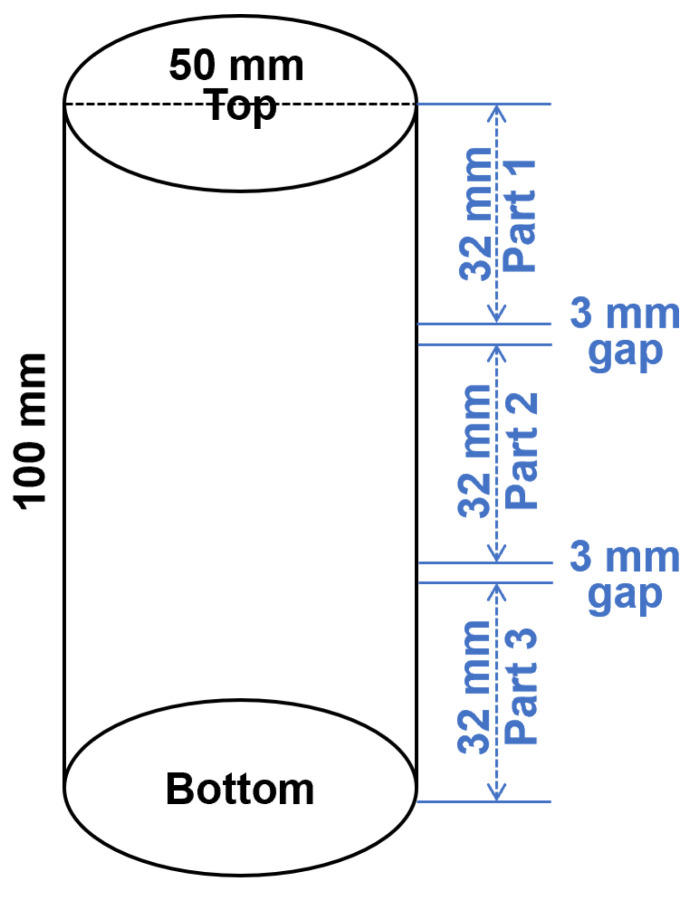
Schematic diagram of the geopolymer cylinder cutting procedure. The 3 mm gaps were reserved for the sample loss during cutting.

After ageing, photos of geopolymer specimens were taken to show the appearance of efflorescence products formed on the surface. The efflorescence products were scraped from the surface and collected to conduct the X-ray diffractometry (XRD) analysis by using the PANalytical X’Pert3 Powder (Malvern, UK) with Cu Ka radiation at a scanning rate of 10°/min from 10° to 80° 2θ. The cylinders with 100 mm height were cut into three slices with 32 mm height. From top to the bottom of cylinder, the slices were denoted as Part 1/2/3, as show in Figure 3. The aged geopolymer slices were dried at 105 °C to achieve a constant initial mass (less than 0.1% mass change within 24 h), and then immersed in water for more than 24 h to achieve a constant final mass. The difference between the initial mass and final mass was calculated to analyze the water absorption capacity.

The compressive strengths of the slices were tested using a mechanical testing machine. Each part had three replicates tested for obtaining the average strength value. The fractured samples were collected and used for measuring the alkali leaching, pore structure and microstructure of geopolymers. 

To measure the content of free alkalis in the geopolymer, the fractured samples were crushed to obtain granular samples with 2.0 ± 0.5 mm diameters, and were mixed with water in a 1:50 solid/water mass ratio. After 24 h of leaching, the pH value of the supernatant was tested by using a pH analyzer (Leici, Shanghai, China). To analyze the pore structure of the granular samples, a nitrogen adsorption measurement was conducted by using a Micrometritics TriStar II 3020 instrument with Barret–Joyner–Halenda model (Norcross, GA, USA). The fractured samples with a 10.0 ± 2.0 mm diameter were also collected, impregnated with low-viscosity epoxy resin and polished by hand with SiC paper. After gold coating, the backscattered electron (BSE) characterization was conducted by using an FEI Quanta FEG 250 scanning electron microscope (SEM, OR, USA) with a 15 kV accelerating voltage.

## 3. Results and Discussion

### 3.1. Efflorescence

#### 3.1.1. Visual Observation

**Figure 4:**
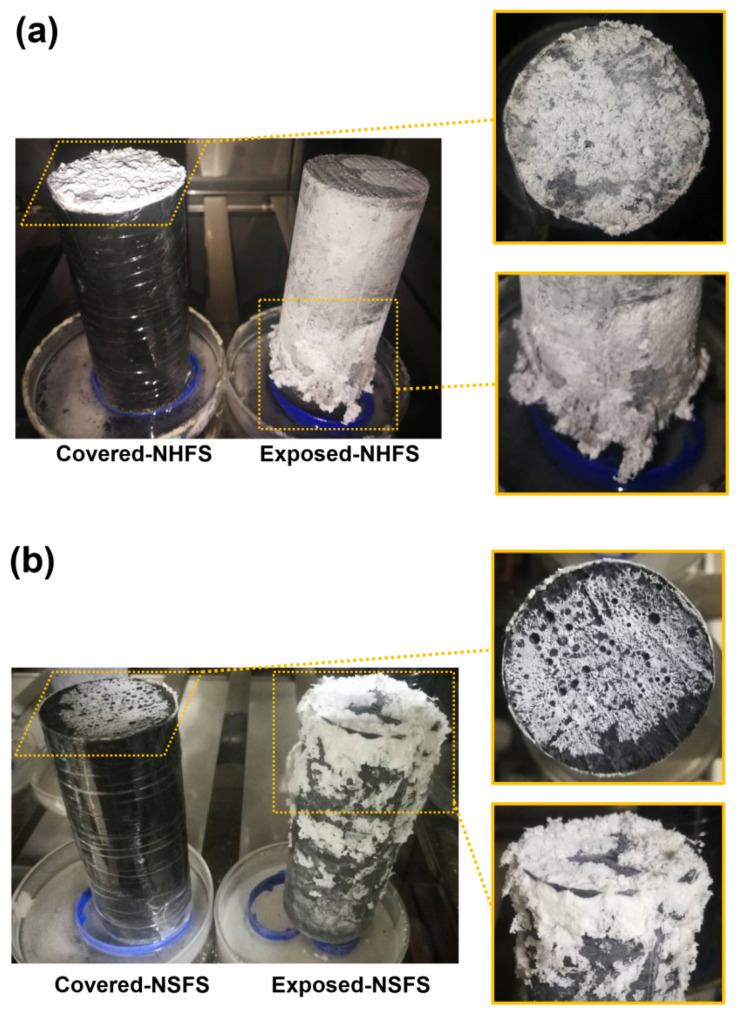
Efflorescence of covered and exposed geopolymer cylinders: (**a**) prepared with NaOH (NHFS) and (**b**) sodium silicate activators (NSFS).

Figure 4 shows the visual observation of efflorescence formed on the surface of covered and exposed NHFS and NSFS geopolymer cylinders. For the covered cylinders, the efflorescence only forms on the top surface. The drying occurred on the top surface induces the moisture movement from the bottom up to the top of the cylinder, transporting a large amount of free alkalis to the top surface to promote the efflorescence formation. More efflorescence products are formed on the top surface of covered NHFS than the covered NSFS. The soluble silicates in the activator seem to restrict the moisture transportation in the pore structure. The geopolymers activated with sodium silicate generally have more compact pore structures than the geopolymers activated with sodium hydroxide [17,18,19]. This is helpful in slowing down the movement of free alkalis in the matrix.

The efflorescence products were formed on the top and around the curved surface of exposed NHFS and NSFS cylinders. It is interesting to note that the efflorescence products were only formed on the lower half of the NHFS geopolymer cylinder. Zhang et al. [9] attributed this phenomenon to the subflorescence occurring at the drying front in the middle of the geopolymer cylinder. The capillary absorption capacity present in the pore structure moves the moisture up to the drying line in the half of cylinder. In comparison, more efflorescence products are formed on the upper half of NSFS geopolymer cylinder. This is probably due to the higher capillary absorption capacity developed in the NSFS, which moves the moisture to a higher height and consequently raises the drying line in the cylinder.

#### 3.1.2. XRD

**Figure 5:**
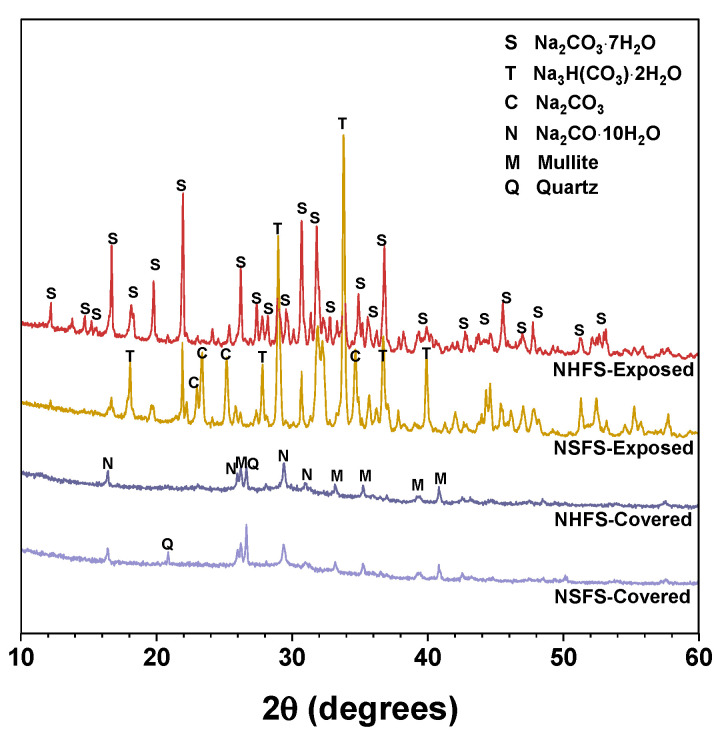
XRD patterns of the efflorescence products scraped from the surface of geopolymer samples. Phases identified: Heptahydrate (S), Na_2_CO_3_·7H_2_O, PDF No. 76-1108; Trona (T), Na_3_H(CO_3_)_2_·2H_2_O, PDF No. 29-1447; Sodium carbonate (C), Na_2_CO_3_, PDF No. 86-302; Natron (N), Na_2_CO_3_·10H_2_O, PDF No. 15-800; Mullite (M), Al_5.65_Si_0.35_O_9.175_, PDF No. 82-1237; Quartz (Q), SiO_2_, PDF No. 74-1811.

Figure 5 shows the XRD patterns of efflorescence products scraped from the surface of covered and exposed NHFS and NSFS samples. Natron (Na_2_CO_3_·10H_2_O) is the main efflorescence product formed on the top surface of covered geopolymer samples. Minor amounts of quartz and mullite are also observed in the efflorescence products. The efflorescence probably induces the structure degradation of geopolymers. Some broken geopolymer pieces are mixed in the efflorescence products during the scraping collection process, so the quartz and mullite are derived from the fly ash remnants in geopolymer. The efflorescence products scraped from the exposed geopolymer samples are mainly composed of heptahydrate (Na_2_CO_3_·7H_2_O), trona (Na_3_H(CO_3_)_2_·2H_2_O) and sodium carbonate (Na_2_CO_3_), which exhibit markedly higher intensity reflections in the XRD patterns than the natron observed in the covered samples [4]. Previous works indicated that the phase evolution of efflorescence was influenced by a variety of factors, such as pore solution compositions and moisture movement in the geopolymer [8,9]. When compared with the heptahydrate and trona formed on the exposed NHFS, more sodium carbonate is formed on the exposed NSFS [20].

#### 3.1.3. Alkali Leaching

**Figure 6:**
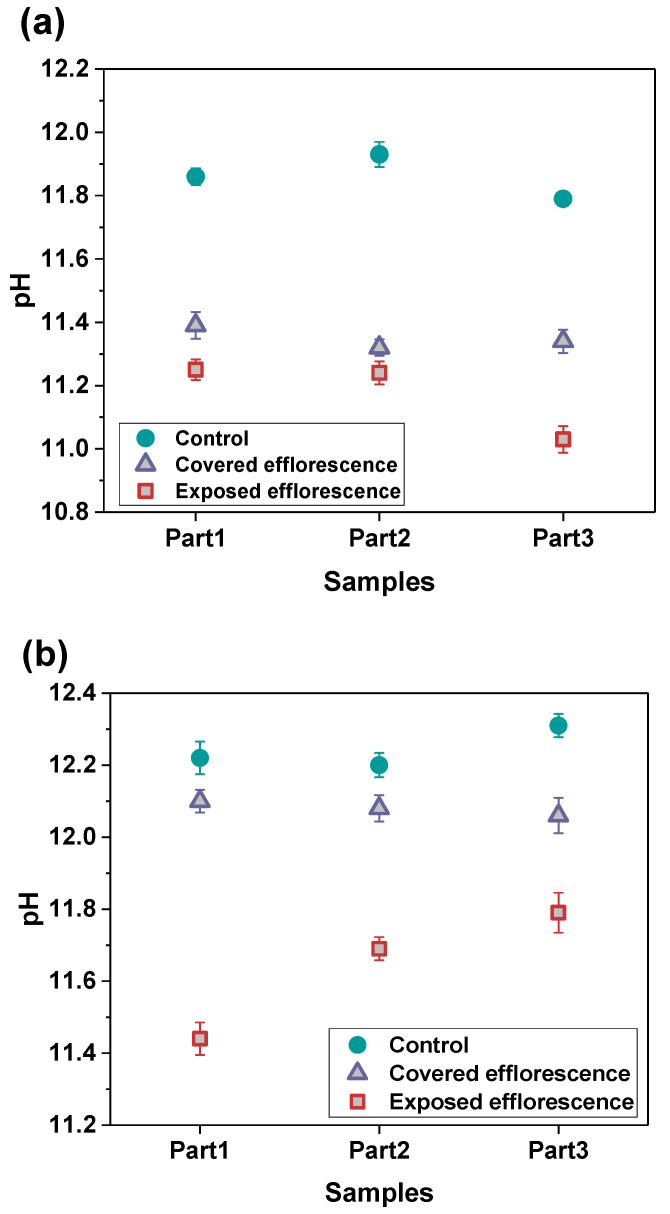
pH values of the leachates of (**a**) NHFS and (**b**) NSFS samples.

Figure 6 shows the alkalis leaching from the NHFS and NSFS geopolymer pastes. The pH values of three parts samples in the control NHFS are in the range of 11.79–11.93, which are lower than 12.20–12.31 in the control NSFS. This suggests that the soluble silicates addition in the activator improves the capacity for retaining alkalis in the geopolymer matrix [21,22]. Higher alkalinity is observed in the control samples than the covered and exposed samples. This confirms the alkali leaching resulted from efflorescence degradation. The pH values of covered samples are higher than the exposed samples. The plastic film covered on the curved surface of the cylinder reduces the contact area with CO_2_ in the air, and consequently slows down the efflorescence and alkali consumption. The pH values of covered NHFS samples are in the range of 11.32–11.39, which is lower than the 12.06–12.10 of the covered NSFS samples. This agrees well with the more intensive efflorescence observed on the top surface of the covered NHFS cylinder (Figure 4). Consistent with the rapid efflorescence formed on the lower half of exposed NHFS cylinder, the pH value of the part 3 sample is lower than the other parts. In comparison, there is a sharp decrease in the pH values from the lower to the upper parts of the exposed NSFS cylinder. This indicates that the free alkali transportation in the exposed NHFS cylinder is clearly distinct from that which occurred in the exposed NSFS cylinder.

### 3.2. Physical Properties and Microstructure Evolution

#### 3.2.1. Compressive Strength and Water Absorption

**Figure 7:**
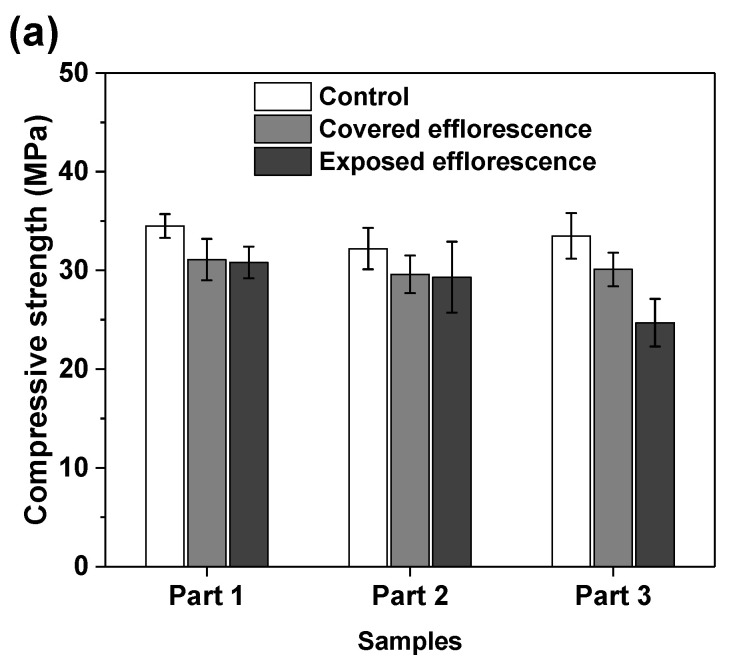

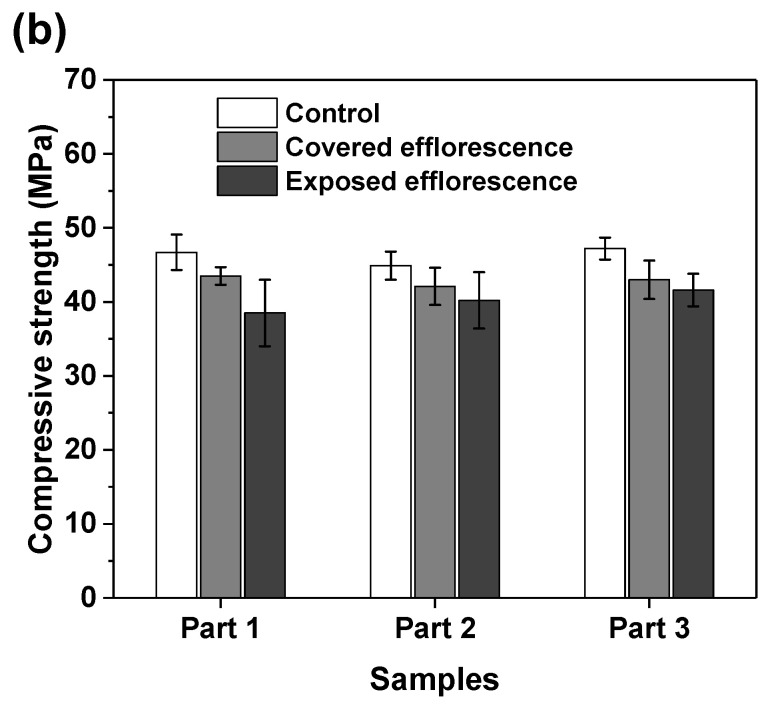
Compressive strengths of (**a**) NHFS and (**b**) NSFS samples.

Figure 7 shows the effects of efflorescence on the compressive strength loss of geopolymer samples. The strengths of control NHFS samples are in the range of 32.2–34.5 MPa, which are lower than the 44.9–47.2 MPa of control NSFS samples. Under the same alkalinity, the soluble silicates in sodium silicate activator lead to a higher extent of geopolymerization than the sodium hydroxide activator [23,24]. The covered NHFS and NSFS samples after efflorescence ageing exhibit slightly lower strengths (29.6–31.1 and 32.1–42.5 MPa) than the control samples. The alkali leaching reduces the reaction extent of the geopolymer. Further reductions in compressive strengths are observed in the exposed samples. It should be noted that the part 3 sample has lower strengths (24.7 MPa) than the part 1 and part 2 samples (30.8 and 29.3 MPa) in the exposed NHFS. In comparison, a slight decrease in strengths from 41.6 to 38.5 MPa is observed for the samples from part 3 to part 1 in the exposed NSFS. Such an observation is consistent with the pH results shown in Figure 6. Zhang et al. [9] observed that the drying front was present in the fly ash-based geopolymer cylinder after efflorescence ageing, and the subflorescence generated the crystallization pressure beneath the drying line. The capillary absorption capacity developed in the pore structure of the NHFS geopolymer can only drive the moisture to the height of drying front, which is in the middle of the cylinder. The part 3 sample beneath the drying line in NHFS is more susceptible to the structure degradation. The NSFS cylinder probably exhibits a higher capillary absorption capacity to drive the moisture from the bottom to the top of cylinder, so there is no drying line observed in the NSFS cylinder. Due to the intensive efflorescence formed on the top and upper half of cylinder, the part 1 sample in NSFS exhibits a higher strength loss.

**Figure 8:**
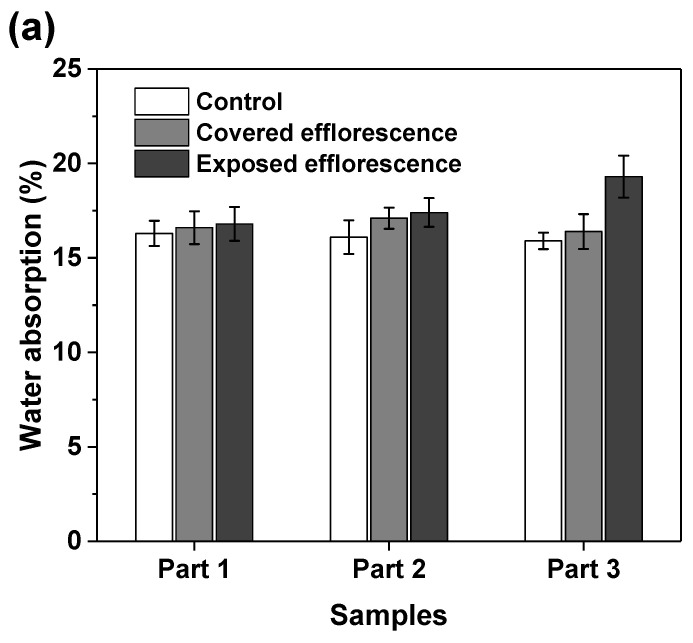

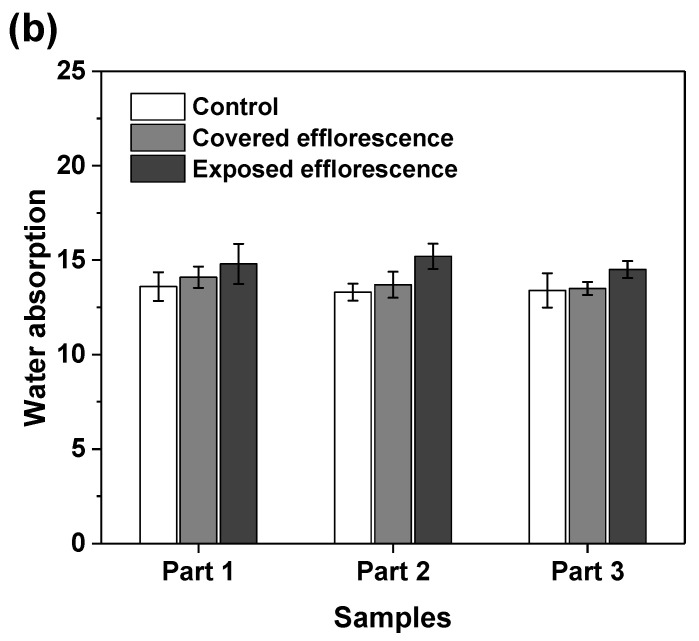
Water absorption of (**a**) NHFS and (**b**) NSFS samples.

Figure 8 shows that the efflorescence increases the water absorption of geopolymers. This supports the speculation that the subflorescence occurred in the matrix results in pore structure degradation to increase the porosity [9]. The NSFS samples have lower water absorption than the NHFS samples, because the soluble silicates in the activator promote the formation of C-A-S-H gel phase to refine the pore structure [25].

#### 3.2.2. Microstructure

**Figure 9:**
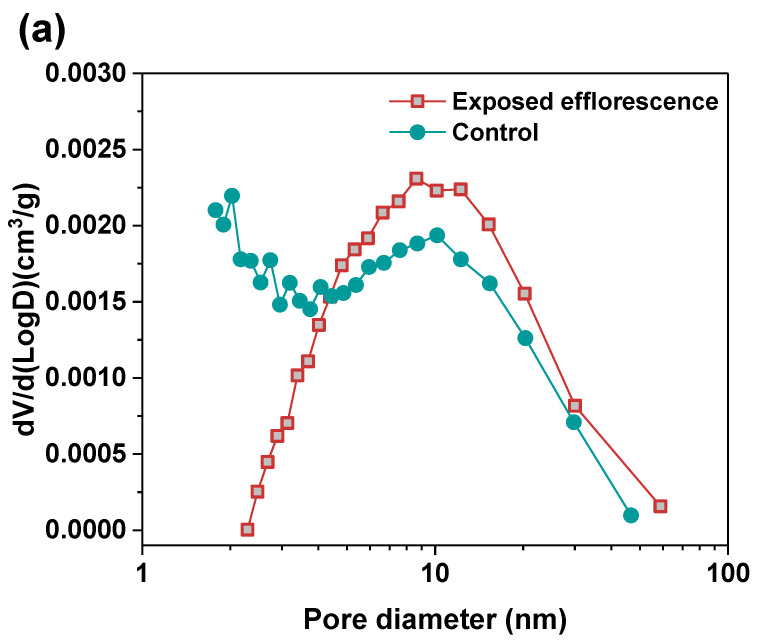

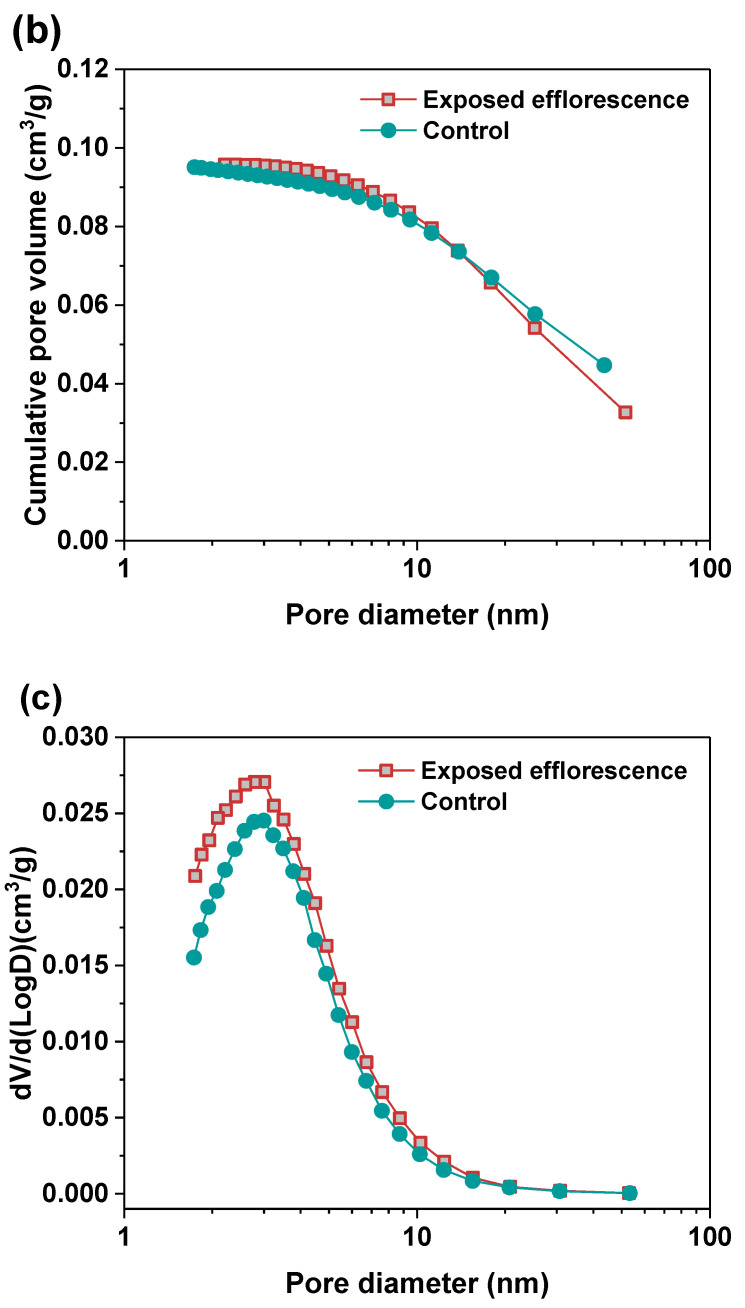

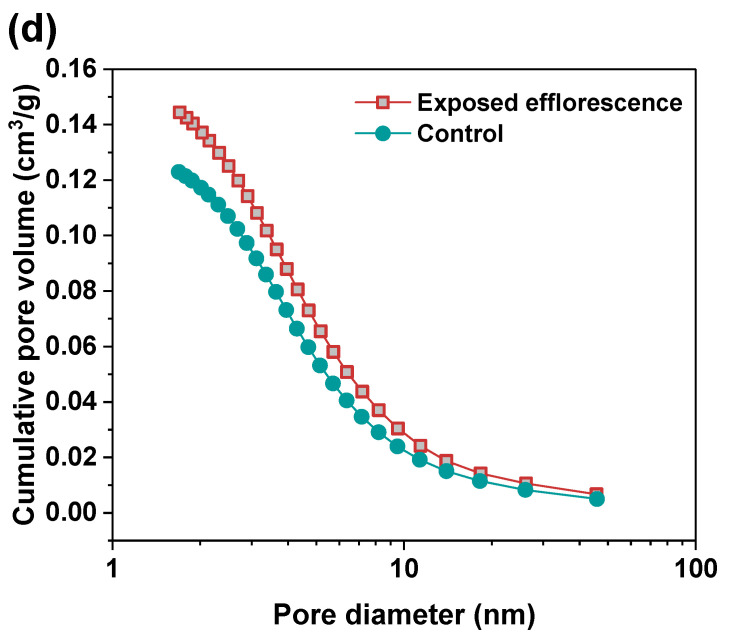
Pore size and cumulative pore volume distribution curves of control and exposed (**a**,**b**) NHFS and (**c**,**d**) NSFS samples.

Nitrogen adsorption method was used to analyze the pore structure differences between the part 3 sample in exposed NHFS and part 1 sample in exposed NSFS. Figure 9 shows the pore size and cumulative pore volume distribution curves of the control and exposed NHFS and NSFS samples, and the relative pore structure parameters are tabulated in Table 2. Although NHFS samples have lower cumulative pore volumes than NSFS samples, the average pore diameters of NHFS samples are higher than those of NSFS samples (Table 2). This suggests that most of the pores in NHFS samples are large capillary pores (10–50 nm), while most of the pores in NSFS samples are medium capillary pores (2–10 nm). The soluble silicates in activators promote geopolymerization. The pore structure of NSFS has a higher tortuosity and lower permeability than that of NHFS [17]. 

After exposed efflorescence ageing, the samples have slightly higher cumulative pore volumes and average pore diameters than the control samples. Such observations are consistent with the water absorption results. The subflorescence induces pore structure degradation. When compared with the control sample, the efflorescence that occurred in part 3 of NHFS reduces the medium capillary pore volume mainly distributed in the range of 1–3 nm, while enhances the proportion of large capillary pores at around 10 nm (Figure 9a). For the part 1 sample in NSFS, the proportion of medium capillary pores at around 3 nm is enhanced by the efflorescence ageing. This suggests that the more refined capillary pore network formed in the exposed NSFS geopolymer provides a higher capillary absorption capacity to promote the moisture transportation in the binder, which moves the free alkalis to the upper half of cylinder to participate in the efflorescence precipitation. In comparison, the lower capillary absorption capacity developed in the less refined pore structure of the exposed NHFS geopolymer can only drive the moisture and free alkalis to the middle of cylinder. The moisture transportation is confronted with the drying front present in the upper half of cylinder [9].

**Figure 10:**
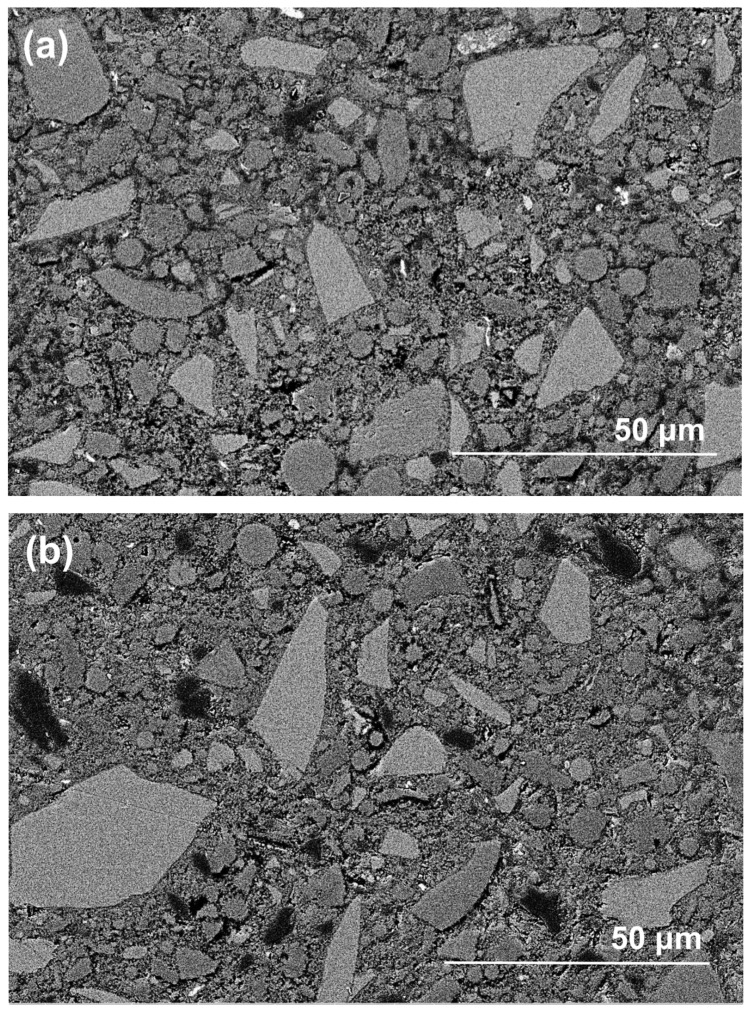

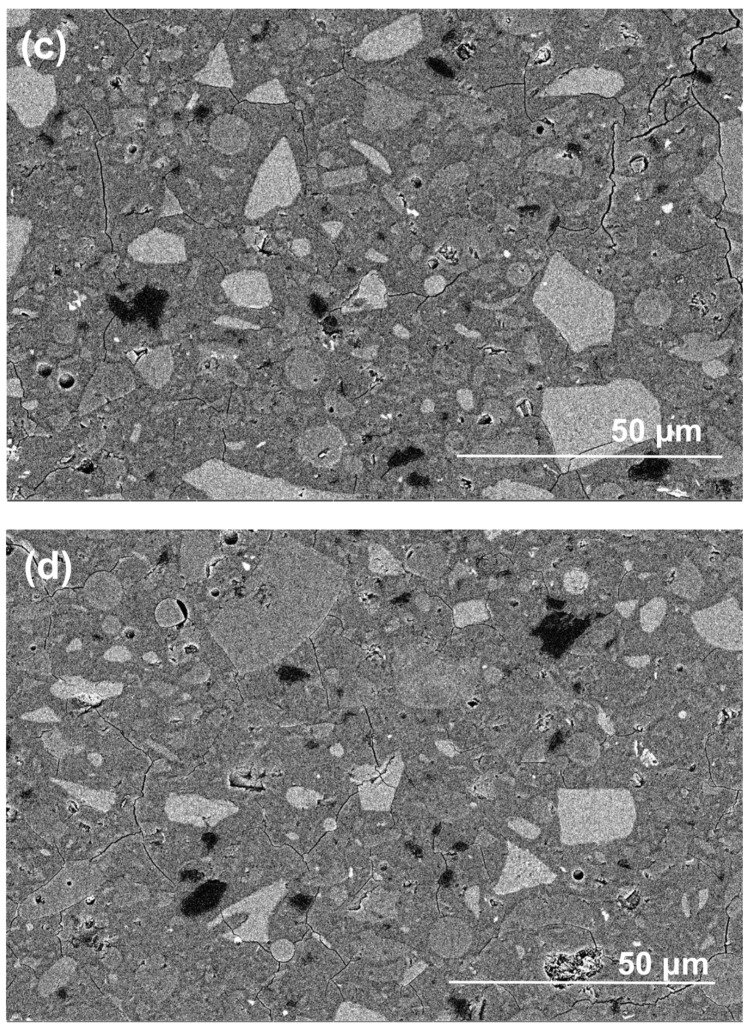
Backscattered electron (BSE) images of (**a**,**b**) part 3 in exposed NHFS and (**c**,**d**) part 1 in exposed NSFS after efflorescence ageing.

Figure 10 shows the morphologies of polished cross-sections in exposed NHFS and NSFS samples after efflorescence ageing. The geopolymer matrix is mainly composed of light-grey angular slag remnants, grey spherical fly ash remnants and grey hydration products [26]. In addition, the black areas distributed in the matrix should be assigned to the pores filled with epoxy resign during the impregnation procedure [27]. Although some microcracks are observed in the NSFS, more pore areas are distributed in the NHFS. It is generally accepted that the geopolymer binder prepared with sodium silicate activator usually exhibits higher extent of reaction and higher drying shrinkage strain than the system prepared with NaOH activators [28]. This seems to be one of the major reasons why microcracks develop in the NSFS sample. Consistent with water absorption and nitrogen adsorption analyses, the NHFS has a more porous microstructure than the NSFS. Moreover, the NHFS is more susceptible to the efflorescence structure degradation.

#### 3.2.3. The Relationship between Moisture Transportation and Efflorescence

**Figure 11:**
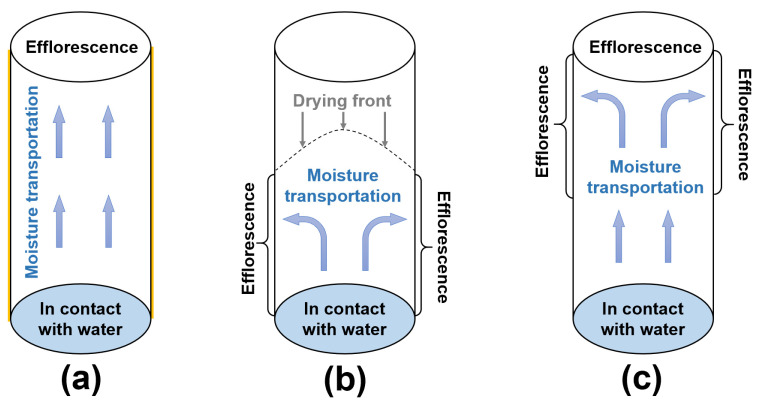
(**a**–**c**) Schematic diagram of moisture transportation in the geopolymer cylinders after different curing regimes.

In this work, the moisture transportation and efflorescence in the fly ash/slag geopolymers should be categorized into three types, which are shown in Figure 11:

(1) For the cylinders covered with plastic film, the capillary suction drives the moisture and free alkalis from the bottom to the top surface of cylinder (Figure 11a). Without drying occurring around the curved surface of cylinder, the more porous structure in NHFS results in higher capillary absorption capacity and more rapid efflorescence than the compact NSFS.
(2) For the cylinders without cover, the capillary pores network determines the moisture transportation in the cylinder. The drying front is observed in the NHFS sample with larger capillary pores. The capillary suction can only drive the moisture to the middle of cylinder (Figure 11b). The efflorescence products are mainly formed on the lower half of cylinder. The NSFS sample with more medium capillary pores has a higher capillary absorption capacity, which can drive the moisture and efflorescence to the top surface of cylinder (Figure 11c). No drying front is observed in this sample.

## 4. Conclusions

This work investigated the relationship between moisture transportation and efflorescence in the sodium hydroxide- or sodium silicate-activated fly ash/slag geopolymers. The geopolymer cylinders were cured in drying conditions (20 ± 1 °C, 60 ± 2% RH) for 28 days with the bottom in contact with water to accelerate efflorescence. The efflorescence products formed on the surface of the cylinder are sodium carbonate hydrates, which are mainly composed of natron, heptahydrate, trona and sodium carbonate. The alkali leaching of geopolymers results in compressive strength loss. The crystallization pressure generated from efflorescence seems to be another reason for the pore structure degradation and water absorption increase in the geopolymers.

When the curved surface of a geopolymer cylinder is covered with plastic film, the efflorescence products are mainly formed on the top surface. The capillary suction drives the moisture and free alkalis from the bottom to the top surface of cylinder. More intense efflorescence is observed on the geopolymer cylinders without surface cover. Efflorescence products are mainly formed on the lower half of uncovered geopolymer cylinders activated with sodium hydroxide. A low capillary absorption capacity developed in the pore structure can only drive the moisture to the height of drying front, which is in the middle of cylinder. In comparison, more efflorescence products are formed on the upper half of uncovered geopolymer cylinders activated with sodium silicate. The more compact pore structure has a higher capillary absorption capacity to transport the free alkalis from the bottom to the upper half so there is no drying line observed in the cylinder.

## Figures and Tables

**Table 1:** Chemical compositions of fly ash and slag as measured by X-ray fluorescence (XRF) (wt.%). Loss on ignition at 1000 °C represented by LOI.

Materials	SiO_2_	Al_2_O_3_	CaO	MgO	Fe_2_O_3_	Na_2_O	K_2_O	TiO_2_	LOI
Fly ash	52.05	33.30	3.80	1.35	3.72	0.54	1.46	1.30	1.06
Slag	30.34	15.46	38.16	7.45	0.40	0.48	0.17	0.49	1.03

**Table 2:** Pore structure parameters of the control and exposed geopolymers.

Samples	Curing Regimes	Medium Capillary Pores 2–10 nm (cm^3^/g)	Large Capillary Pores 10–50 nm (cm^3^/g)	Cumulative Pore Volume (cm^3^/g)	Average Pore Diameter (nm)
Part 3 in NHFS	Efflorescence	0.0162	0.0796	0.0958	18.06
	Control	0.0168	0.0783	0.0951	16.02
Part 1 in NSFS	Efflorescence	0.1202	0.0242	0.1444	4.36
	Control	0.1037	0.0192	0.1229	4.28
